# A Case of Chondroblastoma in the Medial Condyle of the Femur Treated With an Intra-Articular Approach via the Intercondylar Fossa

**DOI:** 10.1155/cro/9978301

**Published:** 2025-02-05

**Authors:** Masato Fumoto, Tomoaki Mori, Tsuyoshi Mizuma, Satoshi Kamio, Sayaka Yamaguchi, Naofumi Asano, Shu Kobayashi, Robert Nakayama

**Affiliations:** Department of Orthopaedic Surgery, Keio University School of Medicine, Tokyo, Japan

## Abstract

Adequate bone curettage is crucial for treating epiphyseal chondroblastomas. However, achieving adequate curettage while minimizing damage to the articular cartilage is challenging. For lesions at the center of the distal femoral epiphysis, curettage using an intercondylar approach may have better oncological and functional outcomes than the conventional extra-articular approach from the wall of the epiphysis. We herein present a case of a 22-year-old male patient with a chondroblastoma of the femoral medial condyle close to the intercondylar fossa. Intercondylar curettage was performed at the site of the insertion of the posterior cruciate ligament (PCL). Maximal knee flexion allowed for good exposure of the lesion, and a direct approach to the tumor led to adequate curettage. Careful suturing of the PCL and postoperative care resulted in sufficient joint stability. The patient showed no signs of local recurrence or osteoarthritic changes at his 16-month follow-up. The intercondylar approach could be a surgical technique worth considering for the treatment of chondroblastoma in the distal femoral epiphysis, particularly for lesions located near the intercondylar fossa.

## 1. Introduction

Chondroblastoma is a rare benign bone tumor commonly found in the epiphyses of long bones and occurs mainly in adolescents and young adults [1]. It is treated using bone curettage, with optional complementary adjuvant therapy. The reported local recurrence rates range from 3% to 32% [1–5] indicating adequate curettage to be essential. However, surgical procedures can cause complications, such as postoperative osteoarthritis due to articular cartilage damage [6, 7].

For lesions in the distal femur, conventional surgical approaches include extra-articular curettage from the medial or lateral wall of the epiphysis [8–10]. Meanwhile, for lesions in the middle two-quarters of the distal femoral epiphysis, better outcomes have been reported with the intercondylar approach [11].

In this report, we describe a case of chondroblastoma located near the intercondylar fossa in the medial condyle of the distal femur that was treated effectively with intra-articular curettage via the insertion site of the posterior cruciate ligament (PCL).

## 2. Case Presentation

**Figure 1:**
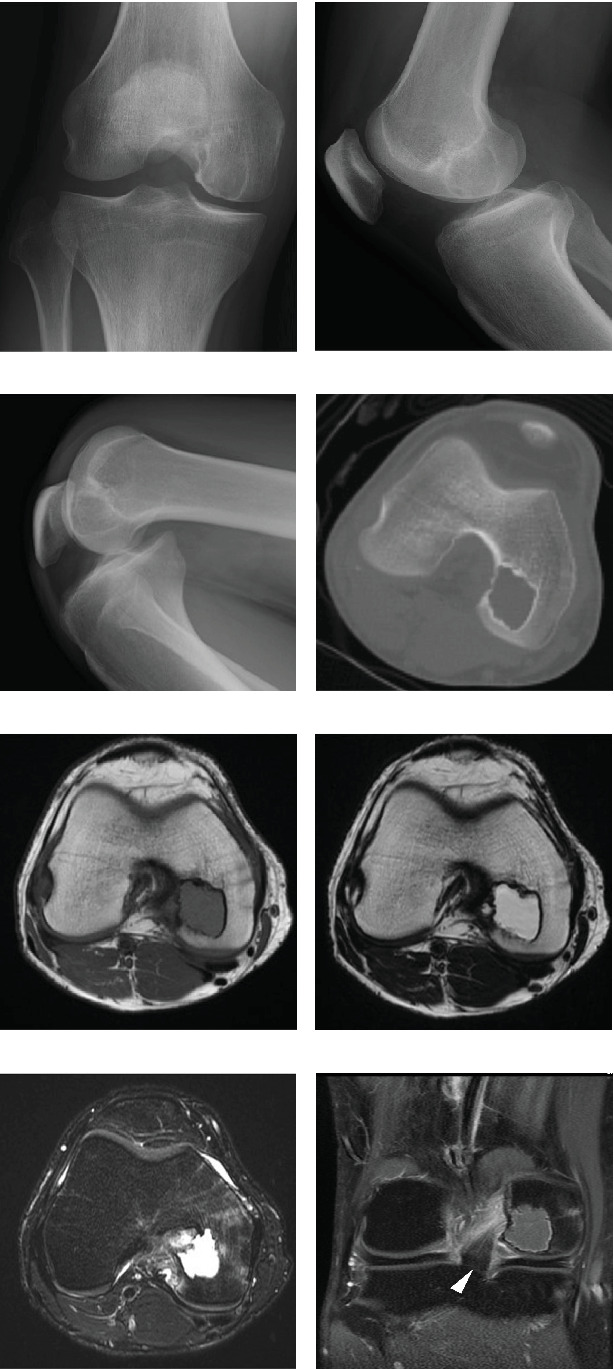
Frontal (a) and lateral (b) views of radiographs showing a well-demarcated radiolucent lesion at the intercondylar side of the medial condyle. (c) Maximal flexion of the knee advanced the lesion anteriorly, providing good view in our surgical field. (d) Axial view of the computed tomographic image showing an epiphyseal lesion with a sclerotic border. An intercondylar approach provided better access to the lesion than a conventional extra-articular approach. Axial view of the magnetic resonance image showing an epiphyseal lesion with low signal intensity on the T1-weighted image (e) and high signal intensity on the T2-weighted image (f). (g) The surrounding bone marrow showed high intensity with the fat-suppressed T2-weighted image. (h) Coronal view of the contrast-enhanced fat-suppressed T1-weighted image showing the tumor at the medial condyle and near the insertion site of the posterior cruciate ligament at the intercondylar fossa.

A 22-year-old man presented with moderate pain in his right knee that had persisted for 7 months. Physical examination revealed no restriction in the range of motion (ROM) or signs of tenderness or swelling related to the knee. All laboratory data were within normal limits. Radiography revealed a well-demarcated radiolucent lesion in the medial condyle (Figures 1(a) and 1(b)) that moved anteriorly with maximal knee flexion (Figure 1(c)). Computed tomography (CT) showed an epiphyseal lesion with a sclerotic border (Figure 1(d)). Magnetic resonance imaging (MRI) revealed T1 hypointense (Figure 1(e)) and T2 hyperintense (Figure 1(f)) epiphyseal lesions. The surrounding bone marrow showed high intensity with fat-suppressed T2-weighed imaging (Figure 1(g)), suggesting edema, and the tumor margin showed mild contrast enhancement (Figure 1(h)). The lesion was located in the medial condyle, near the PCL insertion site at the intercondylar fossa (Figure 1(h)). Considering the patient's age, tumor location, and radiographic findings, chondroblastoma was suspected. Surgical treatment with bone curettage and grafting, combined with an intraoperative frozen section diagnosis, was planned.

**Figure 2:**
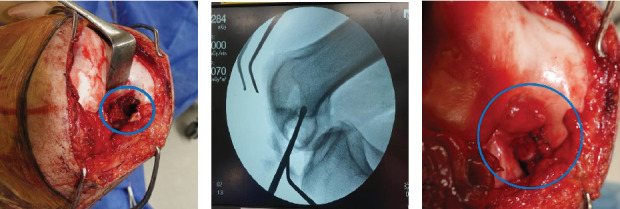
A c-shaped flap door opened for bone curettage (a). The posteromedial bundle of the posterior cruciate ligament has been detached. Bone curettage under intraoperative fluoroscopy was performed using curettes (b). Closure of the flap door after curettage (c).

A longitudinal medial parapatellar incision was made under general anesthesia. Subsequently, the medial patellar retinaculum and joint capsule were incised, allowing the patella to be dislocated laterally. The knee was held in the maximally flexed position to expose the intercondylar fossa. The posteromedial bundle of the PCL was detached from the insertion site, and a C-shaped flap door for bone curettage was opened using an osteotome (Figure 2(a)). After confirming absence of signs of malignancy in the frozen section, bone curettage under intraoperative fluoroscopy was performed using curettes (Figure 2(b)). The inner wall of the lesion was excised using high-speed burring. Finally, the lesion was packed with *β*-tricalcium phosphate, and the flap door was sutured and closed (Figure 2(c)). The posteromedial bundle of the PCL was securely sutured to the synovium of the original insertion site using an absorbable suture. Intraoperatively, it was confirmed that the flap door and sutured PCL remained stable throughout knee flexion from 0° to 90°.

The site of the bone defect was such that it could come into contact with the tibia during knee flexion; thus, early flexion and weight-bearing were restricted. Damage to the PCL was also considered during cautious postoperative care. The patient underwent 2 weeks of non–weight-bearing with knee immobilization at full extension. ROM up to 90° of flexion was allowed after 2 weeks, free ROM was permitted at 8 weeks, and weight-bearing was allowed at 4 weeks.

Pathological findings showed an increased number of osteoclast-like giant cells in the cartilage matrix, and immunostaining for H3K36M showed positive results [12]. Therefore, the patient was diagnosed with chondroblastoma.

**Figure 3:**
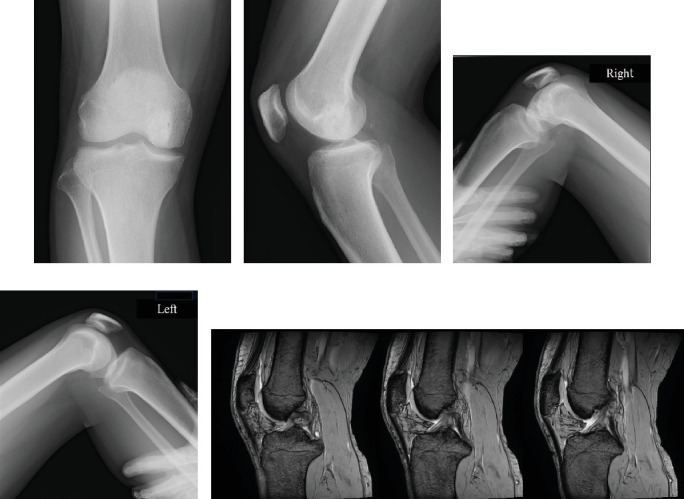
Frontal (a) and lateral (b) views of radiographs showing no signs of arthritic change or collapse of the femoral articular surface at 16 months' follow-up. PCL stress radiographs did not show any signs of increased posterior tibial translation of the affected right knee (c) compared to the left knee (d). Sagittal view of the magnetic resonance image (T2-weighted image) confirmed that the continuity of the PCL was well preserved (e).

The patient showed no signs of local recurrence, pain, joint instability, or restricted ROM 16 months after surgery. Radiography showed no signs of arthritic changes or collapse of the femoral articular surface (Figures 3(a) and 3(b)). PCL stress radiographs did not show any signs of increased posterior tibial translation of the affected right knee (Figure 3(c)) compared to the left knee (Figure 3(d)). MRI also confirmed that the continuity of the PCL was well preserved (Figure 3(e)).

## 3. Discussion

Achieving adequate curettage while preserving the articular cartilage and growth plate is challenging for surgeons when treating chondroblastoma. This dilemma is exemplified by the surgical approach used to treat chondroblastomas of the femoral head. An extra-articular transcervical approach with subtrochanteric entry is aimed at accessing the tumor without damaging the articular surface. However, the poor exposure of the tumor and a narrow pathway for curettage result in local recurrence rates, which are as high as 40%–50% [4, 13]. In contrast, a direct intra-articular approach through the cervical neck or articular cartilage allows for good exposure and easier curettage. Some studies have reported no local recurrences when these direct approaches were applied [4, 13–15]. Considering these studies, we believe that direct access and good exposure of the lesion are beneficial for the treatment of chondroblastoma in the distal femur.

Previous studies have shown that a direct approach to the epiphysis has been favored by several surgeons when treating lesions in the knee joint [8–10, 16, 17], with local recurrence rates as low as 2.8% [8]. These studies have mainly described conventional extra-articular approaches that access the tumor from the wall of the epiphysis. However, a cohort study of 30 children with lesions involving the middle two-quarters of the distal femoral epiphysis showed that an intercondylar approach may result in lower recurrence rates [11]. In our case, the tumor was located in the medial condyle, near the insertion site of the PCL at the intercondylar fossa. Therefore, an intercondylar approach through the insertion site of the PCL provided shorter access, better exposure, and adequate curettage than a conventional extra-articular approach (Figure 1(d)). This approach was made possible by maximal knee flexion, as it advances the lesion anteriorly into an accessible position in our operative field (Figure 1(c)). It is also worthy to point out that in our case, we did not use any chemical intraoperative adjuvant therapy. Previous study using this intercondylar technique has used hydrogen peroxide after the curettage procedure [11]. The usage of adjuvant therapy is still a controversial topic, but our patient has shown no signs of recurrence in our 16-month follow-up. This implies the fact that adequate curettage is indeed the main factor in preventing recurrences and that the intercondylar approach is an optimal technique for achieving sufficient tumor resection.

The overall rates of postoperative osteoarthritis in patients with chondroblastoma have been reported to be up to 38% [3, 6]. However, when focusing on the knee joint, a case series of 36 patients reported a rate of 2.8% [8]. Liu et al. stated that a good blood supply to the distal femur and proximal tibia could reduce secondary osteoarthritis rates in the knee joints [8]. Moreover, chondroblastoma in the knee joint can be accessed directly without penetrating the weight-bearing area of the articular cartilage, for example, through the medial or lateral wall of the epiphysis [8–10, 16, 17], thus reducing the risk of osteoarthritis. For tumors in the middle two-quarters of the distal femoral epiphysis, a cohort study showed that an intercondylar approach may further reduce the risk of knee joint degeneration [11]. This implies that performing a curettage procedure with good exposure of the lesion not only can improve recurrence rates but can also reduce unnecessary damage to the articular cartilage. Although we only observed the patient for 16 months and further follow-up is required, our patient showed no signs of radiographic osteoarthritic changes or knee joint pain.

Growth disturbances are also an issue in patients undergoing surgery with open epiphyseal plates. Huang et al. reported the rate of premature closure of the physis to be 11.9% [17], and Xiong et al. reported the affected limb to be 18.47 ± 7.22 mm shorter than the contralateral side [7]. Interestingly, a patient's age is a well-known factor associated with local recurrence rates [3, 17]. Surgeons' concern about damaging the epiphyseal plates in younger patients leads to inadequate curettage [3]. In the present case, the patient was skeletally mature. Therefore, aggressive curettage and high-speed bur was possible without the fear of postoperative growth disturbance.

A disadvantage of our approach is that the posteromedial bundle of the PCL must be detached. This may be a risk factor for knee instability. Jamshidi et al. have used nonabsorbable suture and a bone tunnel technique to repair the PCL. However, in contrast, we have chosen to suture the PCL to the synovium using absorbable suture. Fortunately, the patient showed no clinical signs of knee joint instability or pain at 16-month follow-up. PCL stress radiographs also did not show any signs of increased posterior tibial translation (Figures 3(c) and 3(d)), and MRI confirmed that the continuity of the PCL was well preserved (Figure 3(e)).

The PCL consists of two bundles: the anterolateral and posteromedial bundles [18]. For the reconstruction of a ruptured PCL, single-bundle reconstruction repairs the anterolateral bundle, whereas double-bundle reconstruction repairs both bundles [19]. A meta-analysis showed no clinical outcomes that strongly supported the use of the double-bundle technique over the single-bundle technique [20]. The clinical importance of the anterolateral bundle may be greater than that of the posteromedial bundle. By suturing the posteromedial bundle to the original insertion site and applying careful postoperative care, the intact anterolateral bundle may have provided sufficient posterior tibial stability to achieve satisfactory short-term outcomes. However, Jamshidi et al. reported a case with a positive posterior drawer test after surgery, implying the importance of two intact bundles [11]. Further follow-up is required to determine the true effects of injured posteromedial bundles.

In conclusion, we treated a case of chondroblastoma near the intercondylar fossa in the femoral medial condyle using an intercondylar approach with curettage through the insertion site of the PCL. The anterior approach, combined with maximal knee joint flexion, allowed sufficient exposure of the lesion, leading to adequate curettage. In addition to the oncological outcomes, the functional outcomes were satisfactory, confirming the results of a previous study [11]. However, further studies are required to fully elucidate the benefits of this approach.

## Data Availability

All relevant data are provided in the manuscript.

## References

[1] Xu H., Nugent D., Monforte H. L. (2015). Chondroblastoma of bone in the extremities: a multicenter retrospective study. *Journal of Bone and Joint Surgery. America Volume*.

[2] Ebeid W. A., Hasan B. Z., Badr I. T., Mesregah M. K. (2019). Functional and oncological outcome after treatment of chondroblastoma with intralesional curettage. *Journal of Pediatric Orthopedics*.

[3] Muratori F., Scanferla R., Roselli G., Frenos F., Campanacci D. A. (2023). Long term outcome of surgical treatment of chondroblastoma: analysis of local control and growth plate/articular cartilage related complications. *BMC Musculoskeletal Disorders*.

[4] Laitinen M. K., Stevenson J. D., Evans S. (2019). Chondroblastoma in pelvis and extremities-a signle centre study of 177 cases. *Journal of Bone Oncology*.

[5] Sailhan F., Chotel F., Parot R., on behalf of the SOFOP (2009). Chondroblastoma of bone in a pediatric population. *Journal of Bone and Joint Surgery. America Volume*.

[6] Farfalli G. L., Slullitel P. A. I., Muscolo L. D., Ayerza M. A., Aponte-Tinao L. A. (2017). What happens to the articular surface after curettage for epiphyseal chondroblastoma? A report on functional results, arthritis, and arthroplasty. *Clinical Orthopaedics and Related Research*.

[7] Xiong Y., Lang Y., Yu Z. (2018). The effects of surgical treatment with chondroblastoma in children and adolescents in open epiphyseal plate of long bones. *World Journal of Surgical Oncology*.

[8] Liu Q., He H., Yuan Y. (2019). Have the difficulties and complications of surgical treatment for chondroblastoma of the adjoining knee joint been overestimated?. *Journal of Bone Oncology*.

[9] Fitzgerald J., Broehm C., Chafey D., Treme G. (2014). Chondroblastoma of the knee treated with resection and osteochondral allograft reconstruction. *Case Reports in Orthopedics*.

[10] Errani C., Traina F., Chehrassan M., Donati D., Faldini C. (2014). Minimally invasive technique for curettage of chondroblastoma using endoscopic technique. *European Review for Medical and Pharmacological Sciences*.

[11] Jamshidi K., Kargar Shooroki K., Ammar W., Mirzaei A. (2024). Does the intercondylar approach provide a better outcome for chondroblastoma of the distal femur in skeletally immature patients?. *The Bone & Joint Journal*.

[12] Rekhi B., Dave V., Butle A., Dutt A. (2023). Utility of immunohistochemical expression of H3.3K36M and DOG1 in the diagnosis of chondroblastomas: an experience from a tertiary cancer referral center. *Annals of Diagnostic Pathology*.

[13] Strong D. P., Grimer R. J., Carter S. R., Tillman R. M., Abudu A. (2010). Chondroblastoma of the femoral head: management and outcome. *International Orthopaedics*.

[14] Katagiri H., Takahashi M., Murata H., Wasa J., Miyagi M., Honda Y. (2022). Direct femoral head approach without surgical dislocation for femoral head chondroblastoma: a report of two cases. *BMC Surgery*.

[15] Xu H., Niu X., Li Y., Binitie O. T., Letson D. G., Cheong D. (2014). What are the results using the modified trapdoor procedure to treat chondroblastoma of the femoral head?. *Clinical Orthopaedics and Related Research*.

[16] Yang Z., Tao H., Ye Z., Huang X., Lin N., Yang M. (2018). The diagnosis and treatment of tibial intercondylar chondroblastoma. *Clinics (São Paulo, Brazil)*.

[17] Huang C., Lü X. M., Fu G., Yang Z. (2021). Chondroblastoma in the children treated with intralesional curettage and bone grafting: outcomes and risk factors for local recurrence. *Orthopaedic Surgery*.

[18] Arthur J. R., Haglin J. M., Makovicka J. L., Chhabra A. (2020). Anatomy and biomechanics of the posterior cruciate ligament and their surgical implications. *Sports Medicine and Arthroscopy Review*.

[19] Dasari S. P., Warrier A. A., Condon J. J. (2023). A comprehensive meta-analysis of clinical and biomechanical outcomes comparing double-bundle and single-bundle posterior cruciate ligament reconstruction techniques. *American Journal of Sports Medicine*.

[20] Migliorini F., Pintore A., Spiezia F., Oliva F., Hildebrand F., Maffulli N. (2022). Single versus double bundle in posterior cruciate ligament (PCL) reconstruction: a meta-analysis. *Scientific Reports*.

